# Women in Railway Service as Viewed from a Surgical Standpoint; Pertinent to the World War

**Published:** 1918-01

**Authors:** E. O’Neill Kane

**Affiliations:** Kane, Pa., First Lieutenant, M.O.R.C.


### WOMEN IN RAILWAY SERVICE AS VIEWED FROM A SURGICAL STANDPOINT., PERTINENT TO THE WORLD WAR.

**BY E. O'Neill Kane, M.D., F.A.C.S., Kane, Pa.,**

First Lieutenant, M.O.R.C.

The demands of war are forcing into railway service, as into all other commercial activities, the gentler sex in order that men, their natural protectors, may meet their "sociologic fate." It is not within the scope of this brief paper to even touch upon the question of woman's right to enter upon vocational activities formerly those of men, her adaptability, or her physiological, pathological and psychological differences in construction. She has already proven her ability to do any thing that a man can do; and her brilliant achievements in professional life, her mechanical ingenuity, as an artisan, and her faithful devotion to duty, have proven her the mistress of the situation to such an extent that it is unnecessary for me to emphasize the fact. I will confine my remarks merely to the surgical aspect of her case as she enters upon a new sphere of labor—that in which brawn and muscle as well as brain and dexterity must subject her to those traumatisms to which men principally were formerly subjected, wherein, right or wrong, she will ultimately excel. The deplorable fact that women must inevitably come to serve in each and every position wherein male railroad employees have heretofore been placed can be prophesied with reasonable certainty if the war continues. Let us consider, therefore, wherein the railway surgeon will find it necessary to change his prophylaxis, his operative procedures, and his after treatment to meet their especial requirements.

Prophylactically speaking, we must seek as rapidly as possible to reconstruct, partially at least, to build up and masculinize women employees in order to range them or a par with men in the same vocations. If we do not do this, not only will they enter upon their new duties more or less handicapped and inefficient, but they will lay themselves open to many accidental injuries which by suitable instruction and physical training might have been avoided. Therefore, I would suggest that women applying for work of the heavier sort be first examined for physical fitness much as are enlisted men for the army, and that when accepted they be, like those in our training camps, put under physical training and given instruction in hygiene for a sufficient period to bring their constitution and musculature up to the highest standard; their training to be somewhat like that pursued in a Y. M. C. A. gymnasium, with especial attention devoted to the trapeze bar, rope climbing, rings, etc., for the development of the upper extremities, and jumping and running to increase agility. Further, it should be incumbent upon their employers in every department to see that these women continue to carry on properly a standardized system of calisthenics, should their daily duties not in themselves prove sufficient. They should be obliged to conform to the same, or equally rational, regulations regarding clothing as men, both as a protection against the weather and in order to prevent their garments being caught in machinery, between cars, etc. They should be prevailed upon to cut their hair. Rings, bracelets and necklaces, and other adornments which are liable to hamper freedom of motion or become entangled in running gear, must be discarded. Corsets, high-heeled shoes, and similar irrational female eccentricities must be abandoned while on duty. Tea and coffee should be abstained from. Fortunately, the enervating influence of tobacco seldom proves a handicap to women.

There should be some consideration given to the age limit of women. There need not be any objection to girls as young as sixteen entering active service (they mature earlier than men), but healthy women after forty-five on the other hand, usually lose vigor and have a tendency to obesity, an undue amount of adipose tissue being depositied about the obdomen and hips which it is difficult to remove except by such a protracted training as is impractible. They are thus rendered too clumsy for active service where agility is required.

The pelvic diseases peculiar to women demand much less atention than many gynecologists would lead us to suppose. Tangible inflamamtory pelvic disorders due to venereal disease, should, of course, disqualify; also marked fibroid degeneration of the uterus with a tendency to hemorrhage, fibroid tumors, ovarian cysts, etc., as, of course, should pregnancy. Mere uterine and ovarian displacements however, even in prolapsus uteri if not complete, cystocele and rectocele, have proven compatible with vigorous and prolonged physical exertion, and, indeed, are better borne and often partially corrected by the change from sedentary habits. The same truth applies to dysmenorrhea, functional or organic, from which so many unmarried women suffer. It often disappears entirely during active physical training.

''Safety first'' should be carefully taught women as well as men, though they will need it less as they are naturally prudent, and, therefore, less prone to injury. By the above outlined physical training, together with the natural superior quickness of action and keener perception of women, railroad injuries will be found less frequent than where men are employed; yet they will sometimes, of course, occur. Let the surgical operator, therefore, consider how their traumatisms, when met with, will be worthy of peculiar consideration, as also wherein their treatment shall be different from that of men. Women's anatomical framework is lighter of construction than that of men. Her bones, on the other hand, are somewhat denser and more elastic. So, fractures do not occur as readily under equal strain; there is less tendency to comminution, and the open compound fracture is less likely to have been infected, as clothing and skin is usually much cleaner. Visceral injuries of all sorts are less apt to prove fatal, the suppleness or resiliency (the springy elasticity) of their viscera rendering their rupture less probable. The blood-vessels and heart of older subjects usually bear traumatism and strain better than in men of the same age because of their more near approach to normality, the tobacco and liquor habits being seldom met with as vascular degenerators. Except in married women, syphilis is seldom found as a disposing factor toward arterial degeneration. Hemorrhage is far better borne by women than by men, probably on account of their physiological ability to recuperate from the menstrual depletion.

Nerve injuries require no special mention save that their repair is more easily effected, from the fact that dissections of every sort are more readily carried out on the less bulky tissues of women. The question of shock is a very serious one. Women bear pain vastly better than men. Phpsiologically they are thus constructed in order to enable them to endure the pains of accounchment. There is here, however, too often a deceptive quasi appearance of tranquility in women suffering to the point of agony, because of their natural stoical submission to pain. In such cases late shock is liable to occur as a result of over-endurance. On the whole, however, I believe women will be found less often affected by shock than men, and to a lighter degree. They certainly endure tedius operative procedure of all sorts better. Anesthesia under all anesthetics is more rapidly and quietly induced and more safely conducted with women, as a rule, than with men. It has been suggested that the more simple life led by women, their more temperate habits and freedom from tobacco and alcoholic addiction account for this. I believe, however, that there is some additional factor. It might be supposed that the tendency to hysterical manifestations correctly ascribed to many women would contrast most unfavorably, in the management of surgical cases, with the more phlegmatic masculine character. We must bear in mind, however, that but few, if any, truly hysterical women will undertake the class of labor under consideration. Such as do will soon fall out of the ranks through obvious incapacity. The surgeon will, therefore, have no more hysteria in the operating room from these women than from men; but he must not overlook the greater impressibility of these more highly and delicately organized members of society, and should treat them more tenderly and also show due appreciative compassion for their injuries. Sympathy is not wasted upon suffering humanity in any case, but it is especially grateful to women and does much toward tiding them through their surgical ordeals; like fine china they cannot bear rough treatment well.

In the after treatment of surgical cases women are but little different from men, save that they are more exacting of attendants, more complaining (faultfinding, perhaps), and in less of a hurry to get out of bed. Yet their greater desire to get back to work and avoid losing their jobs is some offset to their natural love of ease and the little creature comforts afforded to a convalescent. And here a word of caution is not amiss relative to the more imaginative temperament of women exerting a prejudicial influence upon complete recovery from a phychological standpoint. It is to the enforced stay in bed that most of the imagined permanent disabilities, such as railway-spine and other obscure nerve disturbances, owe their origin. The surgeon should, therefore, be on the watch for all tentative questions or suggestion made by his female patient relative to such condition, and be prepared with suitable replies and explanations regarding supposed abnormal sensations.

After recovery, if disabilities prove permanent, it is gratifying to observe that women most readily and rapidly adapt themselves to the altered circumstances. This fact is greatly in their favor. They handle artificial limbs better than men do, though they may complain more if they fail to get a good fit, and wear them longer without getting them out of order. If given some form of employment campatible with the crippling undergone they soon show themselves as useful members of society as if physically perfect, and cause their employers little further trouble if pettifogging attorneys can be eliminated.

**Conclusions:**

Women can, if necessary, fill any and every branch of railway service. They will do better work than men, their superior mentality making up for inferiority in bulk and brawn. (A preliminary intensive calisthenic training is essential to their safety and efficiency). They are better surgical risks, and cause the surgeon less trouble on the operating table and less anxiety during convalescence than their masculine competitors. Greater care should be taken to prevent their bringing damage suits against their railroad company, owing to their psychological susceptibility to suggestion. —International Journal of Surgery.

